# Clinical course and demographic insights into suicide by self-poisoning: patterns of substance use and socio-economic factors

**DOI:** 10.1007/s00127-024-02750-x

**Published:** 2024-09-24

**Authors:** Stefanie Geith, Maja Lumpe, Johannes Schurr, Sabrina Schmoll, Christian Rabe, Armin Ott, Raphael Stich, Michael Rentrop, Florian Eyer, Tobias Zellner

**Affiliations:** 1 Department of Clinical Toxicology, Klinikum Rechts der Isar, TUM School of Medicine and Health, Technical University of Munich, Ismaninger Str. 22, 81675 Munich, Germany; 2 Staburo GmbH, Munich, Germany; 3 Clinic and Policlinic for Psychiatry and Psychotherapy, Klinikum Rechts der Isar, Technical University of Munich, Munich, Germany; 4 kbo-Inn-Salzach Clinic, Wasserburg am Inn, Germany

**Keywords:** Suicide, completed, Suicide, attempted, Self-poisoning, Epidemiology, High-risk medication

## Abstract

**Purpose:**

To analyze whether sociodemographic characteristics influence the substance choice and preclinical and clinical course of suicidal poisoning.

**Methods:**

This was a retrospective single-center study in patients hospitalized due to suicidal poisoning and who received at least one psychiatric exploration during their inpatient stay. Patients’ sociodemographic, anamnestic, preclinical, and clinical parameters were analyzed with respect to sex and age.

**Results:**

1090 patients were included, 727 (67%) were females, median age was 39 years (min–max: 13–91) with 603 (55%) aged 18–44 years. 595 patients (54.8%) ingested a single substance for self-poisoning, 609 (59.5%) used their own long-term medication. Comparing to males, females preferred antidepressants (*n* = 223, 30.7%, vs *n* = 85, 23.4%; *p* = 0.013) and benzodiazepines (*n* = 202, 27.8%, vs *n* = 65, 17.9%; *p* < 0.001); males more often used cardiovascular drugs (*n* = 33, 9.1%, vs *n* = 34, 4.7%; *p* = 0.005) and carbon monoxide (*n* = 18, 5.0%, vs *n* = 2, 0.3%; *p* < 0.001). Use of Z-drugs (*n* = 1, 1.7%, to *n* = 37, 33.3%; *p* < 0.001) and benzodiazepines (*n* = 4, 6.9%, to *n* = 33, 29.7%; *p* = 0.003) increased with age (< 18 to > 64 years), while use of non-opioid analgesics (*n* = 23, 39.7%, to *n* = 20, 18.0%; *p* < 0.001) decreased. Average dose of substance in patients > 64 years was 12.9 ± 18.4 times higher than recommended maximum daily dose (compared to 8.7 ± 15.2 higher in those aged < 18 years; *p* < 0.001). Males more often required intensive care (*n* = 150, 41.3%, vs *n* = 205 females, 28.2%; *p* < 0.001).

**Conclusion:**

These results underline the complexity of (para-)suicidal poisonings and identify potential measures for their prevention, such as restricting access and better oversight over the use of certain substances.

**Supplementary Information:**

The online version contains supplementary material available at 10.1007/s00127-024-02750-x.

## Introduction

Self-poisoning ranks second after hanging among the most frequently used suicide methods in Europe in terms of completed suicides [1, 2]. Among suicide attempts, deliberate self-poisoning represents the most frequent method [3, 4]. Recent analyses highlight an increasing frequency of self-poisoning in several countries, including Germany, the United Kingdom, Italy, the United States, Australia, Brazil, Egypt, and Brazil [5–11] and the COVID-19 pandemic exacerbated this trend [12–15]. Suicidal behavior varies with regard to socio-economic as well as cultural factors, but also according to different living circumstances and places of residence, and therefore partly shows considerable international and regional differences [16]. Hatzitolios described variations in substance choice in rural versus urban areas [17], however, our research group did not detect national preferences regarding the substances chosen in an earlier study [18]. In the same study, paracetamol ended up in fourth place among the most frequently used individual substances [18], while in other studies it was the most frequently used substance [19, 20]. Moreover, benzodiazepines, which are usually among the three most popular substances [19–23], ranked fourth among the used substance groups identified in our earlier survey [18]. These observed differences stress the need for the development of specific preventive measures, which take into consideration the individual, but also national or regional peculiarities.

Since many studies on suicides are carried out by psychiatric centres, the emphasis is often on psychiatric and not on somatic issues. In contrast, we focused our research on understanding the socio-demographic characteristics, preclinical factors, the clinical course and, in particular, toxicological aspects of self-poisoning. Exploration of these aspects of suicide is possible due to the structure of our department which encompasses the intensive care unit (ICU), internal medicine and toxicology. This ensures comprehensive patient follow up from presentation in the emergency room through (intensive) medical and specific toxicological therapy to discharge or onward transfer to psychiatry. The advantages of this unique research setting enabled us—after analyzing the characteristics and predictive factors of severe or fatal suicide outcome and the socio-demographic and psychiatric profile of patients hospitalized due to deliberate self-poisoning in previous publications [24, 25]—to address on the following questions in this paper: (i) do anamnestic or socio-demographic factors influence suicidal behavior, the (pre-)clinical course or substance choice in patients with intentional self-poisoning? (ii) do any sex- or age-group-specific characteristics exist with regard to substance choice or suicidal behavior?

## Methods

### Study design and setting

In this retrospective single-centre study, electronic records of patients treated in the Department of Clinical Toxicology at the Klinikum rechts der Isar of the Technical University of Munich, Germany. during the period from 01.01.2012 to 31.12.2016 were analyzed. Patients with self-poisoning based on suicide-related behavior (SRB), who attempted or completed suicide, and who were explored by a board-certified psychiatrist at least once during the inpatient stay in non-fatal cases were included in the study. SRB was defined as self-harm, suicide attempt and suicide, according to the definition of Silverman et al. [26]. (Para-)suicidal intent was diagnosed during the psychiatric consultation or assumed in case of fatal outcome, for example when a suicide note was found, or the relatives confirmed patient’s wish to die. Patient records were examined with regard to sociodemographic, anamnestic, preclinical and clinical parameters. A written informed consent was not obtained from patients due to the retrospective character of the study. The study was approved by the Ethic Committee of the University Hospital (No. 270/16s), and it was conducted in compliance with the Declaration of Helsinki.

### Data collection

Patient data were extracted from the electronic medical records and transferred into a Microsoft Access database. In the analysis, only the demographic information from the initial SRB was considered for patients with multiple clinical admissions. Age-grouped analyses involved categorizing patients into four groups: <18 years, 18–44 years, 45–64 years, and > 64 years. Stratification of patients into four groups was further done according to the rescue time until hospital admission, with intervals defined as < 1 h, 1–3 h, 3–6 h, and > 6 h.

Information on the type and quantity of substances used was based on the information provided by the patient, information from outpatient services, details provided by companions, and results from toxicological analyses. In addition to the usual ingestion of medications, the recording encompassed other substances and non-pharmacological items, such as the ingestion of alkalis, batteries, paper clips or mushrooms for example  or the inhalation of carbon monoxide for example. The ingested substances were systematically classified into various pharmacologically defined groups. Non-opioid analgesics included Ibuprofen, naproxen, diclofenac, acetylsalicylic acid, acetaminophen, and metamizole, while the category “z-drug” comprised the medicines zolpidem and zopiclone. Illegal amphetamines, such as methylenedioxymethamphetamine and methamphetamine, were categorized as “illegal drugs”, while amphetamines prescribed for therapeutic purposes, such as methylphenidate, for the treatment of attention deficit hyperactivity disorder, were assigned to substance category “other drugs”. For each patient, up to eight substances were recorded, with each substance group documented only once per patient. Substance doses were reported by the patients themselves, by others, or estimated from empty packaging blisters. The aim was to obtain a uniform measure of overdoses of different substances, the multiple of the maximum daily dose (MMDD) for ingested substance was calculated based on an overdose or underdose of the substance-specific maximum daily dose (MDD). The determination of the MDD was based on the product information. The MDD used for the calculations are listed in Online Resource Table 1.

**Table 1:** Patient sociodemographic characteristics with respect to sex

Characteristic	Total *n* = 1090	Male *n* = 363	Female *n* = 727	*p*-Value
Age				
Median age (range)	39.0 (13.0, 91.0)	42.0 (15.0, 88.0)	38.0 (13.0, 91.0)	0.089
Age group				
< 18 years	58 (5.3)	13 (3.6)	45 (6.2)	0.023
18–44 years	603 (55.3)	188 (51.8)	415 (57.1)	
45–64 years	318 (29.2)	125 (34.4)	193 (26.5)	
> 64 years	111 (10.2)	37 (10.2)	74 (10.2)	
Family situation				
Single	494 (49.2)	183 (54.3)	311 (46.6)	0.032
Married	299 (29.8)	91 (27.0)	208 (31.1)	
Divorced	137 (13.6)	46 (13.6)	91 (13.6)	
Widowed	49 (4.9)	8 (2.4)	41 (6.1)	
Living separately	26 (2.6)	9 (2.5)	17 (2.5)	
Missing	85	26	59	
Job situation				
Employed	329 (36.2)	107 (35.7)	222 (36.4)	< 0.001
Jobless^a^	149 (13.7)	72 (24.0)	77 (12.6)	
Retired	142 (15.6)	45 (15.0)	97 (15.9)	
Pupil/student/trainee	138 (15.2)	31 (10.3)	107 (17.5)	
Self-employed	39 (4.3)	17 (5.7)	22 (3.6)	
Not working^b^	66 (7.3)	10 (3.3)	56 (9.2)	
On sick leave	22 (2.4)	8 (2.7)	14 (2.3)	
Asylum seekers	14 (1.5)	8 (2.7)	6 (1.0)	
Disability pension	8 (0.9)	0 (0.0)	8 (1.3)	
Incapacitate	3 (0.3)	2 (0.7)	1 (0.2)	
Missing	180	63	117	
Currently working				
Yes	368 (40.4)	124 (41.3)	244 (40.0)	0.754
No	542 (59.6)	176 (58.7)	366 (60.0)	
Missing	180	63	117	
Living environment				
With partner	374 (37.1)	111 (32.8)	263 (39.3)	0.006
Alone	338 (33.6)	129 (38.2)	209 (31.2)	
With parents/family/relatives	172 (17.1)	47 (13.9)	125 (18.7)	
Residential facility/assisted living	58 (5.8)	20 (5.9)	38 (5.7)	
Residential community	28 (2.8)	11 (3.3)	17 (2.5)	
Asylum seeker housing	22 (2.2)	14 (4.1)	8 (1.2)	
Homeless	15 (1.5)	6 (1.8)	9 (1.3)	
Missing	83	25	58	83
Residence				
Large city > 100,000 inhabitants	733 (68.3)	230 (64.1)	503 (70.4)	0.128
Medium city 50,000–99,999 inhabitants	7 (0.7)	3 (0.8)	4 (0.6)	
Small town 20,000–49 999 inhabitants	77 (7.2)	24 (6.7)	53 (7.4)	
Rural town 5000–19,999	187 (17.4)	73 (20.3)	114 (16.0)	
Rural area < 4999	69 (6.4)	29 (8.1)	40 (5.6)	
Missing	17	4	13	17
Parenthood				
Yes	451 (45.4)	129 (39.8)	322 (48.1)	0.017
No	543 (54.6)	195 (60.1)	348 (51.9)	
Missing	96	39	57	96

Data are in *n* (%) unless otherwise noted. Due to rounding, percentages may not add up to 100%

^a^Jobless denotes patients searching for job

^b^Not working denotes parents on maternal, paternal, or parental leave, stay-at-home spouses, working-age adults living with their parents, individuals in assisted living group or under legal supervision

### Statistical analysis

Descriptive data analysis was performed using SPSS Statistics (version 25, IBM Corp., Armonk, NY, USA) and R (version 3.5.2; R Foundation for Statistical Computing, Vienna, Austria). Continuous variables were summarized as mean ± standard deviation (± SD) and categorical variables as absolute and relative frequencies. Pearson’s Chi-squared test or Fisher’s exact test were used for analysis of nominal and ordinal variables with the sample sizes of five or fewer; interval scaled variables were assessed by Student’s *t*-test. *p*-Values ≤ 0.05 were considered statistically significant; because of the exploratory character of this study, we did not adjust for multiple testing.

## Results

Out of 1287 cases of hospitalizations due to a suspected (para-)suicidal intoxication, 1140 had complete data (Online Resource Fig. 1). Among them, 50 cases of hospitalizations were a second or later admission in the same patient and were excluded. Therefore, 1090 patients were included in the final analysis; their characteristics are show in Tables 1 and 2.

**Fig. 1:**
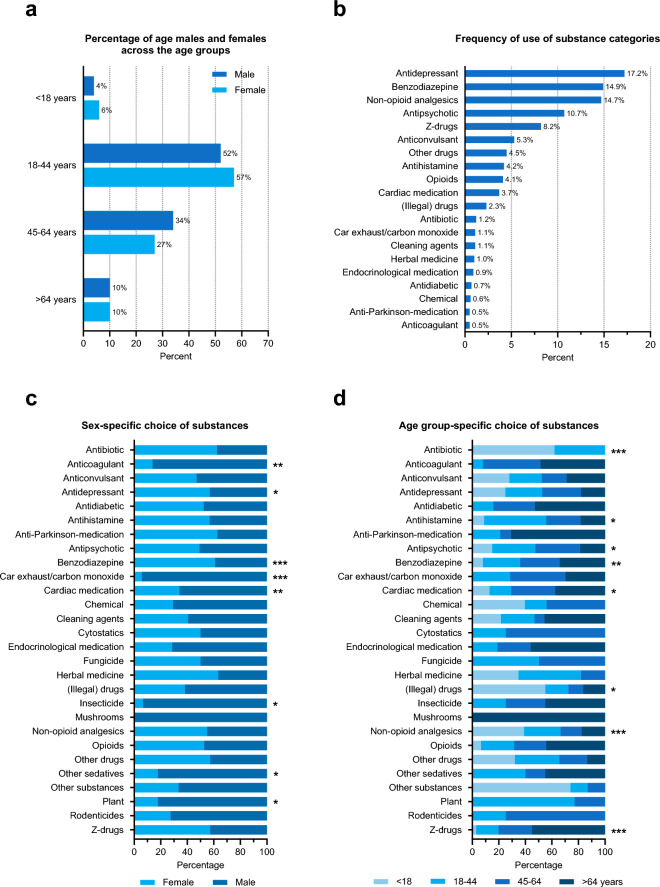
Percentage of males and females across the age groups (**a**) and the frequency of use of substance categories in all patients (**b**). Percentage of sex- (**c**) or age-group-specific (**d**) substance choice. *p < 0.05; **p < 0.01; ***p < 0.001. Each substance category was recorded only once per patient

**Table 2:** Patient sociodemographic characteristics with respect to age-group

Characteristic	Total *n* = 1090	< 18 *n* = 58	18–44 *n* = 603	45–64 *n* = 318	> 64 *n* = 111	p-Value
Sex						
Male	363 (33.3)	13 (3.6)	188 (51.8)	125 (34.4)	37 (10.2)	0.023
Female	727 (66.7)	45 (4.1)	415 (57.1)	193 (26.5)	74 (10.2)	
Family situation						
Single	494 (49.2)	57 (100)	354 (63.4)	64 (22.1)	19 (18.8)	< 0.001
Married	299 (29.8)	0 (0)	134 (24.0)	128 (44.3)	37 (36.6)	
Divorced	137 (13.6)	0 (0)	56 (10.0)	67 (23.2)	14 (13.9)	
Widowed	49 (4.9)	0 (0)	2 (0.4)	17 (5.9)	30 (29.7)	
Living separately	26 (2.6)	0 (0)	12 (2.2)	13 (4.5)	1 (1.0)	
Missing	85	1	45	29	10	
Job situation						
Employed	329 (36.2)	2 (3.6)	221 (45.2)	100 (38.5)	6 (5.7)	< 0.001
Jobless^a^	149 (13.7)	1 (1.8)	90 (18.4)	57 (21.9)	1 (1.0)	
Retired	142 (15.6)	0 (0)	14 (2.9)	35 (13.5)	93 (88.6)	
Pupil/student/trainee	138 (15.2)	50 (89.3)	88 (18.0)	0 (0)	0 (0)	
Self-employed	39 (4.3)	0 (0)	17 (3.5)	19 (7.3)	3 (2.9)	
Not working^b^	66 (7.3)	1 (1.8)	33 (6.7)	30 (11.5)	2 (1.9)	
On sick leave	22 (2.4)	0 (0)	10 (2.0)	12 (4.6)	0 (0)	
Asylum seekers	14 (1.5)	2 (3.6)	12 (2.5)	0 (0)	0 (0)	
Disability pension	8 (0.9)	0 (0)	4 (0.8)	4 (1.5)	0 (0)	
Incapacitate	3 (0.3)	0 (0)	0 (0)	3 (1.2)	0 (0)	
Missing	180	2	114	58	6	
Currently working						
Yes	368 (40.4)	2 (3.6)	238 (48.7)	119 (45.8)	9 (8.6)	< 0.001
No	542 (59.6)	54 (96.4)	251 (51.3)	141 (54.2)	96 (91.4)	
Missing	180	2	114	58	6	
Living environment						
With partner	374 (37.1)	0 (0)	188 (34.6)	144 (47.8)	42 (39.3)	< 0.001
Alone	338 (33.6)	0 (0)	167 (30.8)	115 (38.2)	56 (52.3)	
With parents/family/relatives	172 (17.1)	44 (78.6)	105 (19.3)	19 (6.3)	4 (3.7)	
Residential facility/assisted living	58 (5.8)	6 (10.7)	33 (6.1)	14 (4.7)	5 (4.7)	
Residential community	28 (2.8)	0 (0)	25 (4.6)	3 (1.0)	0 (0)	
Asylum seeker housing	22 (2.2)	6 (10.7)	15 (2.8)	1 (0.3)	0 (0)	
Homeless	15 (1.5)	0 (0)	10 (1.8)	5 (1.7)	0 (0)	
Missing	83	2	66	17	4	
Residence						
Large city > 100,000 inhabitants	733 (68.3)	37 (63.8)	410 (69.7)	208 (65.8)	78 (70.3)	0.468
Medium city 50,000–99,999 inhabitants	7 (0.7)	0 (0)	6 (1.0)	1 (0.3)	0 (0)	
Small town 20,000–49,999 inhabitants	77 (7.2)	7 (12.1)	45 (7.7)	19 (6.0)	6 (5.4)	
Rural town 5000–19,999	187 (17.4)	9 (15.5)	90 (15.3)	69 (21.8)	19 (17.1)	
Rural area < 4999	69 (6.4)	5 (8.6)	37 (6.3)	19 (6.0)	8 (7.2)	
Missing	17	0	15	2	0	
Parenthood						
Yes	451 (45.4)	1 (1.7)	182 (32.5)	198 (70.5)	70 (73.7)	< 0.001
No	543 (54.6)	57 (98.3)	378 (67.5)	83 (29.5)	25 (26.3)	
Missing	96	0	43	37	16	

Data are in *n* (%) unless otherwise noted. Due to rounding, percentages may not add up to 100%

^a^Jobless denotes patients searching for job

^b^Not working denotes parents on maternal, paternal, or parental leave, stay-at-home spouses, working-age adults living with their parents, individuals in assisted living group or under legal supervision

### Sociodemographic data

Patient characteristics according to sex and according to age group are shown in Tables 1 and 2, and Online Resource Table 2, respectively. Median age was 39 years (range 13, 91), 67% (*n* = 727) of the collective were female compared to 33% male patients (*n* = 363, Tables 1 and 2). The group of 18- to 44-year-olds was most frequently represented (*n* = 603; 55%), ahead of the 45- to 64-year-olds (*n* = 318; 29%) and the > 64-year-olds (*n* = 111; 10%, Tables 1 and 2). The smallest age group was the < 18-year-olds (*n* = 58; 5%). Overall, sexes were significantly differently distributed within the four age groups (*p* = 0.023, Tables 1 and 2; Fig. 1a). The relative proportion of females predominated in the two youngest age groups, while the relative proportion of males dominated in the 45–64 age group. The oldest group showed a balanced sex ratio in percentage terms. The age distribution without grouping was similar within the sex groups (*p* = 0.089, Table 1).

Statistically significant differences according to sex and to age were found with regard to family situation, occupational situation, living environment, and parenthood. Females were more often married or widowed, and more often lived with their family or partner (Table 1). The proportion of patients living alone increased with age (Table 2). Patients aged < 18 years or 18- to 44-year-olds mostly lived in communal living arrangements such as shared flats, assisted living or asylum seeker facilities, or lived with parents or relatives. Homelessness was only present in the middle age groups (18–44 and 45–64 years). Current occupation showed no significant differences within the sex groups (*p* = 0.754). Males were more frequently jobless (i.e. searching for job) than females (*n* = 72; 24%, vs *n* = 77; 13%, Table 1). The proportion of females predominated among pupils/students/trainees and those with no occupation. Occupation and family situation yielded age-typical results (Table 2). Almost half of females, and approximately two-thirds of patients in older age groups had children. Residence (size of the city, rural area) did not differ according to sex or age group (Tables 1 and 2). Majority of patients were German (*n* = 323, 65.1%); information on nationality was missing for 594 patients (Online Resource Table 2).

### Anamnestic data

Overall, somatic pre-existing or concomitant cardiovascular, neurological and metabolic diseases were similarly frequent (13.5-13.7%, Online Resource Table 2). Substance abuse was self-reported by 45.5% of patients (Tables 3 and 4).

**Table 3:** Patient anamnestic, clinical and follow-up therapy data with respect to sex

Characteristic	Total *n* = 1090	Male *n* = 363	Female *n* = 727	*p*-Value
*Anamnestic data*				
Abuse				
Nicotine	325 (29.8)	127 (35.0)	198 (27.2)	0.01
Alcohol	101 (9.3)	47 (12.9)	54 (7.4)	0.004
Illegal drugs	70 (6.4)	29 (8.0)	41 (5.6)	0.174
Source of supply				
Own medication	609 (59.5)	183 (55.1)	426 (61.6)	< 0.001
Medicine chest/OTC	138 (13.5)	44 (13.3)	94 (13.6)	
Multiple sources	125 (12.2)	34 (10.2)	91 (13.2)	
Relatives/friends	44 (4.3)	9 (2.7)	35 (5.1)	
Illegally obtained	8 (0.8)	4 (1.2)	4 (0.6)	
Other sources	9 (0.9)	2 (0.06)	7 (1.0)	
No medication	91 (8.9)	56 (16.9)	35 (5.1)	
Missing	66	31	35	66
Trigger factors				
Partner	337 (31.3)	110 (30.8)	227 (31.5)	< 0.001
Family	101 (9.4)	24 (6.7)	77 (10.7)	
Health	83 (7.7)	26 (7.3)	57 (7.9)	
Job	71 (6.6)	29 (8.1)	42 (5.8)	
Social environment	58 (5.4)	18 (5.0)	40 (5.6)	
Loss reaction	36 (3.3)	5 (1.4)	31 (4.3)	
Finance	30 (2.8)	19 (5.3)	11 (1.5)	
Law/justice	23 (2.1)	16 (4.5)	7 (1.0)	
No trigger	338 (31.4)	110 (30.8)	228 (31.7)	
Missing	13	6	7	13
*Clinical data*				
Therapy site				
ICU	355 (32.6)	150 (41.3)	205 (28.2)	< 0.001
IMC	735 (67.4)	213 (58.7)	522 (71.8)	
Number of substances ingested, mean (± SD); median (range)	1.9 (± 1.4); 1.0 (1.0, 13.0)	1.8 (± 1.2); 1.0 (1.0, 7.0)	1.9 (± 1.4); 1.0 (1.0, 13.0)	0.087
MMDD, mean (± SD); median (range)	8.7 (± 12.2); 4.9 (0.2, 109.0)	8.8 (± 11.1); 5.0 (0.2, 100.0)	8.6 (± 12.6); 4.3 (0.2, 109.0)	0.262
Co-ingestion				
Alcohol	338 (31.0)	127 (35.0)	211 (29.0)	0.053
Illegal drugs	16 (1.5)	10 (2.8)	6 (0.8)	0.026

Data are in *n* (%) unless otherwise noted. Due to rounding, percentages may not add up to 100%

*MMDD* multiple of the maximum daily dose

**Table 4:** Patient anamnestic, clinical and follow-up therapy data with respect to age-group

Characteristic	Total *n* = 1090	< 18 *n* = 58	18–44 *n* = 603	45–64 *n* = 318	> 64 *n* = 111	*p*-Value
*Anamnestic data*						
Abuse						
Nicotine	325 (29.8)	18 (31.0)	195 (32.3)	97 (30.5)	15 (13.5)	0.001
Alcohol	101 (9.3)	0 (0)	37 (6.1)	54 (17.0)	10 (9.0)	< 0.001
Illegal drugs	70 (6.4)	1 (1.7)	44 (7.3)	19 (6.0)	6 (5.4)	0.407
Source of supply						
Own medication	609 (59.5)	16 (28.6)	311 (55.6)	209 (69.2)	73 (68.2)	< 0.001
Medicine chest/OTC	138 (13.5)	10 (17.9)	98 (17.5)	27 (8.9)	3 (2.8)	
Multiple sources	125 (12.2)	11 (19.6)	73 (13.1)	29 (9.6)	12 (11.2)	
Relatives/friends	44 (4.3)	10 (17.9)	23 (4.1)	6 (2.0)	5 (4.7)	
Illegally obtained	8 (0.8)	0 (0)	7 (1.3)	1 (0.3)	0 (0)	
Other sources	9 (0.9)	1 (1.8)	4 (0.7)	3 (1.0)	1 (0.9)	
No medication	91 (8.9)	8 (14.3)	43 (7.7)	27 (8.9)	13 (12.1)	
Missing	66	2	44	16	4	
Trigger factors						
Partner	337 (31.3)	4 (6.9)	238 (39.8)	82 (26.2)	13 (12.0)	< 0.001
Family	101 (9.4)	16 (27.6)	51 (8.5)	27 (8.6)	7 (6.5)	
Health	83 (7.7)	1 (1.7)	28 (4.7)	24 (7.7)	30 (27.8)	
Job	71 (6.6)	6 (10.3)	38 (6.4)	26 (8.3)	1 (0.9)	
Social environment	58 (5.4)	7 (12.1)	38 (6.4)	8 (2.6)	5 (4.6)	
Loss reaction	36 (3.3)	0 (0)	9 (1.5)	16 (5.1)	11 (10.2)	
Finance	30 (2.8)	0 (0)	10 (1.7)	18 (5.8)	2 (1.9)	
Law/justice	23 (2.1)	3 (5.2)	10 (1.7)	10 (3.2)	0 (0)	
No trigger	338 (31.4)	21 (36.2)	176 (29.4)	102 (32.6)	39 (36.1)	
Missing	13	0	5	5	3	
*Clinical data*						
Therapy site						
ICU	355 (32.6)	15 (25.9)	180 (29.9)	113 (35.5)	47 (42.3)	0.025
IMC	735 (67.4)	43 (74.1)	423 (70.1)	205 (64.5)	64 (57.7)	
Number of substances ingested, mean (± SD); median (range)	1.9 (± 1.4); 1.0 (1.0, 13.0)	1.7 (± 1.2); 1.0 (1.0, 6.0)	2.0 (± 1.4); 1.0 (1.0, 10.0)	1.8 (± 1.2); 1.0 (1.0, 7.0)	2.0 (± 1.6); 1.0 (1.0, 13.0)	0.109
MMDD, mean (± SD); median (range)	8.7 (± 12.2); 4.9 (0.2, 109.0)	8.7 (± 15.2); 3.5 (0.7, 90.0)	7.3 (± 10.7); 3.7 (0.2, 109.0)	10.4 (± 11.8); 6.7 (0.2, 75.0)	12.9 (± 18.4); 5.3 (0.2, 100.0)	< 0.001
Co-ingestion						
Alcohol	338 (31.0)	7 (12.1)	188 (31.2)	127 (39.9)	16 (14.4)	< 0.001
Illegal drugs	16 (1.5)	2 (3.4)	14 (2.3)	0 (0)	0 (0)	0.004

Data are in *n* (%) unless otherwise noted. Due to rounding, percentages may not add up to 100%

*MMDD*, multiple of the maximum daily dose

Males more often abused nicotine (*p* = 0.01) and alcohol (*p* = 0.004) than females (Table 3). Abuse of nicotine was lowest in patients aged > 64 years (13.5% vs. 30.5–32.3% in other age groups, *p* = 0.001), while patients aged 45–64 years more often abused alcohol (17% vs 0–9% in other age groups, *p* < 0.001, Table 4).

Used substances were predominantly patients´ own continuous medication (*n* = 609; 59.5%), followed by over-the-counter medicines (OTC), from the medicine cabinet (*n* = 138; 13.5%, Tables 3 and 4). Distribution of substance sources within the sex groups also showed significantly different results (*p* < 0.001, Table 3). The sex-specific difference was most remarkable for the substance source “no medication”, with a proportion of 16.9% (*n* = 56) for males versus 5.1% (*n* = 35) for females. The substance source “own medication” was most frequent within each age group and had the highest average age of 43.6 years (Table 4). A tendency towards the substance sources “relatives/friends”, “medicine chest/OTC” and “several sources” was shown within the youngest age group (Table 4).

While almost one third (*n* = 337; 31%) of patients stated a partnership conflict as a trigger for the current suicide(-attempt), almost the same number of patients (*n* = 338; 31%) could not name a trigger factor (Tables 3 and 4). Trigger factors were distributed significantly differently within the sex groups (*p* < 0.001, Table 3). While there were no significant sex-specific differences in the most frequent trigger factor “partner” or “no trigger”, males more frequently named the trigger factors “job”, “finances” and “justice”, while females stated preferentially the trigger factors “loss of a caregiver/pet” and “family problems”. The trigger factors were significantly differently distributed also within the age groups (*p* < 0.001, Table 4). Patients aged < 18 years frequently mentioned family problems, followed by problems in the social environment, while 18 to 44-year-olds frequently mentioned problems with their partner. Within the oldest group, health problems were most often mentioned, followed by conflict with partner, and the loss of a caregiver or a pet.

### Preclinical and clinical data

In 69% of cases (*n* = 659), the rescue service was called by relatives or friends, and more than half of patients had a normal level of consciousness with a GCS of 15 when the ambulance arrived (Online Resource Table 2).

67% of patients (*n* = 735) were monitored and treated in the intermediate care unit (IMC) and 33% (*n* = 355) were admitted to the ICU. Almost 30% of patients stayed in hospital for < 24 h, a comparable percentage of 26% was treated in hospital for > 97 h (Online Resource Table 2). Males were more frequently treated in the ICU and females more frequently in the IMC (*p* < 0.001, Table 3). The necessity of intensive medical therapy increased with age (*p* = 0.025, Table 4).

9% of patients (*n* = 97) received gastrointestinal decontamination with activated charcoal. This was administered by the ambulance service as well as in-hospital per os or via gastric tube after endotracheal intubation (Online Resource Table 2). 11% of cases (*n* = 121) required mechanical ventilation. 16% of patients (*n* = 178) were treated with an antidote (Online Resource Table 2). In addition to the classical antidotes such as flumazenil, naloxone or acetylcysteine, antidotes in the broader sense such as oxygen in the context of carbon monoxide poisoning, physostigmine in case of anticholinergic symptoms or magnesium in case of QTc time prolongation were administrated. Use of vitamin K or prothrombin concentrate in case of intoxication with anticoagulants, insulin-glucose in case of beta-blocker overdose, or ethanol therapy in case of ethylene glycol intoxication were also administered as antidote therapy.

Males had a higher rate of co-ingestion of alcohol during the SRB compared to females (35% vs 29%), although this difference was not significant (*p* = 0.053); co-ingestion of drugs occurred more than twice as often in males than in females (*p* = 0.026, Table 3). In case of co-ingestion of alcohol (*p* < 0.001) or drugs (*p* = 0.004), these substances differed according to age group (Table 4). Co-ingestion of alcohol was found most frequently in the two middle age groups, whereas co-ingestion of drugs was found only in the two youngest age groups.

#### Substance pattern

54.8% of patients (*n* = 595) took only one substance in the context of the suicide attempt (Online Resource Table 2). Mixed intoxications with two or three substances were documented in 22% (*n* = 239) and 12% (*n* = 133) of patients, respectively. Almost 95% of patients used up to three substances. In four cases (0.4%) the number of substances was unknown. On average, 1.9 substances (± 1.4) were used (one patient had a mixed intoxication with 13 single substances, Tables 3 and 4).

Females showed a non-significant tendency towards the use of a higher number of substances than males during suicide attempt (*p* = 0.087, Table 3); no age-group-specific differences in the number of substances used were noted (*p* = 0.109, Table 4).

Antidepressants, benzodiazepines and non-opioid analgesics were chosen most frequently; medications from each of these substance classes were ingested with other agents in approximately two-thirds of cases (Fig. 1b, Online Resource Table 3). For both sexes, the same substance groups dominated, albeit with different preferences. When comparing the substances within the sex groups (Fig. 1c, Online Resource Table 3), it is noticeable that males significantly more often chose carbon monoxide (*p* < 0.001), insecticides (*p* = 0.017), cardiovascular drugs (*p* = 0.005), plants (nutmeg, castor bean or belladonna) (*p* = 0.045), anticoagulants (*p* = 0.008) and other sedatives (*p* = 0.045). Females preferred antidepressants (*p* = 0.013) and benzodiazepines (*p* < 0.001).

With regard the age group, an antibiotic use was documented in 5% and 3% within the two youngest age groups (< 18 and 18-44 years, respectively) while this substance class was not used by older patients (Fig. 1d, Online Resource Table 3). The highest rate of antihistamines and neuroleptics use was in the middle age groups. Use of cardiovascular drugs (*p* = 0.012), benzodiazepines (*p* = 0.003) and Z-drugs (*p* < 0.001) increased with age. The highest rate of drugs and narcotics was in those aged < 18 years at 12% (*n* = 7, *p* = 0.02). Non-opioid analgesics predominated within the two youngest age groups. No differences in opioids use between sex and age groups were observed.

Overall, the average MMDD was 8.7 ± 12.2, with no differences between the sexes (Table 3). However, the MMDD differed between the age-groups (*p* < 0.001) with a highest average value in patients aged > 64 years (12.9 ± 18.4) and lowest in those aged 18–44 years (7.3 ± 10.7, Table 4).

## Discussion

The main findings of our study are that (i) there is an influence of sex on the frequency, mode, and severity of suicidal self-poisonings and (ii) age associates with the patterns of substance choice in suicidal self-poisonings. These findings may be useful for targeted programs of suicide prevention.

The average age of 40.5 years with a 1:2 male: female ratio in this work confirms the results of comparable studies [17, 20, 28, 29]. Similarly as in the UK study [30], the sex ratio converged in the older age groups. 34% of the cohort had a citizenship other than German. Compared to around 26% of the German population with a migration background or with 12% foreigners, the proportion in this study is significantly higher [31]. This may be due to a bias towards a major city in southern Germany with around 1.4 million inhabitants (the predominant enrollment area), with a higher proportion of non-German citizens.

Proportion of jobless patients was significantly higher than the average jobless rate in Germany in the study period (14% vs 6.7%) [32] thus supporting the notion that individuals searching for job are at a higher risk for (para-)suicidal behavior [33, 34].

As in comparable studies [35, 36], most patients presented to hospital within 3 h of substance use.

The substance pattern, with 55% of mono-intoxications and a preferred choice of antidepressants (17%), followed by benzodiazepines (15%) and non-opioid analgesics (15%), is similar to the results of recent studies [20, 37], in which antidepressants were used more frequently than benzodiazepines than in older studies [35, 38]. The tendency towards increased antidepressant vs benzodiazepines use in the context of (para-)suicidal poisoning has been observed in other studies, and it was associated with the increased frequency of antidepressant prescribing [39, 40]. This is supported by the fact that almost 60% of our patients ingested their own long-term medication. Analysis of substance choice revealed a sex-specific pattern with preference for antidepressants and benzodiazepines among females and use of carbon monoxide, chemicals, insecticides, drugs/intoxicants, anticoagulants, cardiovascular drugs, and other sedatives among males. The preferred choice of a pesticide/insecticide [27, 41] or gases, and chemicals [42] by males has already been described and was reconfirmed in this study, in which males were about three times more likely to use non-medical substances for SRB than females (17% vs 5%). Similar to the results reported here, Muheim et al. demonstrated that females more often chose non-opioid-analgesics and antidepressants, while males preferred sedatives. In contrast, a significantly increased use of opioids in males could not be confirmed in our work [43].

In terms of age-specific differences, younger patients tended to use antibiotics, antihistamines, drugs/narcotics, and non-opioid analgesics more frequently than older patients. Preferential use of non-opioid analgesics in the youngest patients was also found in a study by Froberg et al. [44] and could be due to a weaker intention to die, unawareness of the generally wider therapeutic range of this class of drugs (with the exception of acetaminophen), or ease of availability. The elderly were more likely to choose benzodiazepines, Z-drugs, anticoagulants, and cardiovascular drugs, i.e. long-term medications in this age group. Accordingly, we observed the highest frequency of own medication as a source in patients aged > 64 years. Of note, we did not observe differences in opioids use between the age groups, however, prior analysis found that individuals aged 45 to 54 years had a highest rates of suicide deaths with opioid poisoning in US [45].

9% of cases underwent therapeutic interventions, such as the administration of activated charcoal. In other studies, this frequency ranged from 1.5% to about two-thirds of patients [20, 46, 47]. This considerable variability may be a consequence of a limited preclinical availability of activated charcoal, variation in patient compliance, and a limited appropriate time window. Hemodialysis in 2% of cases and antidote therapy in 16% was comparable to a previous report (2% and 18%, respectively) [17].

In a study by Cook et al., the majority of patients were discharged within 24 h [20]. In our study, the length of inpatient stay was longer, as most patients stayed over 24 h. This may reflect a relatively high proportion of psychiatric follow-up therapy in this study, as a seamless transfer may result in a longer stay due to time bridging. Alternative explanation could be that our patients were more severely poisoned with 33% treated in the ICU compared to 1.5% in UK [20], 16% in Israel [28], and 11% in Greece [17]. The rate of ICU treatment in another German study was 61%, almost twice as high as in our study [19]. These different rates of ICU stays may be influenced by fluctuating ICU capacities or internal hospital monitoring strategies. For example, in most hospitals, many patients must be monitored in an ICU after a suicide attempt, even in mild poisoning cases, because adequate monitoring and self-protection cannot be guaranteed on a normal ward. Our department with an ICU and protected IMC, offers the possibility of a seamless “step-up/step-down concept”, which allows monitoring of severely poisoned patients in the ICU and less severe cases in the IMC, thus providing a better individualized monitoring of patients. Given the current shortage of intensive care beds, studies showed that from an internal medicine perspective, most self-poisonings can be adequately and sufficiently treated on an IMC or with supportive therapy by means of monitoring alone [20, 46, 48].

Co-ingestion of alcohol in about one in three patients is in line with the results of comparable studies [20, 38, 46]. Co-ingestion of alcohol predominated among males and in the two middle age groups, which corroborates the results of a similar study [42]. Also, a significantly more frequent co-ingestion of drugs was found in males. Regarding the age group-specific differences, co-ingestion of alcohol among adolescents (< 18 years) was significantly lower in this work (12%) than in a Finnish study, which detected alcohol in about half of the adolescents who committed suicide [49]. Thus, adolescents with a completed suicide appear to differ from the group of young people who attempted suicide in terms of co-ingestion of alcohol.

Alcohol and nicotine abuse predominated among males. Comparably, Mauri et al. found a significantly higher proportion of alcohol dependence (“alcoholism”) in males with (para-)suicidal intoxication [35]. According to a 2021 study, 17.6% of the German population had “harmful” alcohol consumption [50]. In contrast, only 9.3% of patients self-reported the alcohol abuse. Nicotine abuse in 30% of our cohort corresponded to the daily or occasional smoking behavior of 18- to 64-year-old German adults [50]. Illicit substance abuse (6.4%) was higher compared to the general German adult population (2.5%, [50]). However, above-average substance abuse rate is not surprising, as addictive disorders are associated with an increased risk of SRB [51]. The tendency of young males to abuse illicit substances more frequently [50] was hinted in this study (8% males versus 6% females), but without statistical significance.

Considering possible reasons for SRB, partner and family conflicts were common trigger factors, comparable to the findings of Hatzitolios et al. [17]. Males more often reported problems with job, finances, or law or justice as trigger factors, while females more often suffered from the loss of a caregiver or pet. This corresponds to the observations of a study from Hong Kong, which found a predominantly interpersonally triggered parasuicidal behavior in females compared to a personally triggered behavior in males [52]. Interestingly, there were no statistically significant differences in problems in relationship with partner between the sex groups in your study. In contrast, a Turkish study recorded relationship problems more frequently in female than in male suicides [53]. Furthermore, a study investigating psychosocial factors associated with suicidal thoughts in comparison to suicide attempts revealed that relational trauma was a significant risk factor distinguishing males from females [54]. Nevertheless, that study included not only subjects who attempted suicide but also those who reported suicidal ideations, and furthermore, did not distinguish between the means of suicide. In contrast, our study focused specifically on (para-)suicidal poisoning, which could potentially account for a lack relationship problems among triggers differentially reported by females and males. Furthermore, family problems and problems with the social environment were mentioned most frequently by the youngest patients and partner conflicts by those aged 18 to 44, while health problems and “loss of caregiver/pet” were predominantly documented for those aged ≥ 64 years. Similarly, loss of caregivers and associated isolation have been discussed as risk factors for suicide attempts in the elderly [55]. Finally, males had a significantly higher percentage of intensive care treatment. This could be explained by fact that, compared to females, males were more likely to develop a higher severity level of poisoning. In our prior analysis of the present cohort, we demonstrated a lower frequency of parasuicides in men, which is presumably associated with a stronger intention to die [25]. Furthermore, compared to females, males more often used non-medical substances which we found previously to predict severe or fatal course of suicide [24]. Higher lethality of suicidal behavior in males has been demonstrated in past studies not only by choice of method but also within the same method [56, 57].

### Limitations

Our study has several limitations including the retrospective nature, the monocentric setting, and a suspected sampling bias. Patients aged < 18 years were the smallest age group because our center mainly treats adults and only in exceptional cases children. This could be perceived as a bias since distribution of patients into age groups was done according to clinical considerations, but not to ensure a uniform group size. Furthermore, most of patients came from the area of a major German city, which could not be representative for a broader population. Therefore, it can be supposed that frequency of singles living alone as well as use of drugs could be higher than in rural regions.

Since this was a single-center survey of a pre-selected patient population, it can be assumed that many intentional overdoses, also with (para-)suicidal intent, did not lead to a medical presentation due to a barely or slightly symptomatic course. For example, some patients reported having taken drugs in the past with suicidal intent but resuming their usual daily routine a few hours later after a symptom-free interval and without this incident being noticed by anyone. On the other side of the clinical spectrum, severely intoxicated persons who are found dead or do not reach a clinic alive are not included in this work. Overall, a certain selection bias of patients must be assumed.

Information on the ingested substances (in cases without toxicological analysis), trigger factors, and suicide circumstances was often based on incomplete information provided by the patients or obtained during external anamneses. In some cases, the information on the dose of the substances could only be estimated by the paramedics based on empty blisters or medicine boxes found in the rubbish bins. However, there remains considerable uncertainty as even in cases when date of purchase or dispensing was known, this was not necessarily the time of first substance use and thus could lead to dose underestimation. In contrast, in cases when empty blisters were found, it was assumed that all tablets were ingested which could lead to dose overestimation. In certain instances, an indication of quantity was only possible by means of the number of tablets taken without knowledge of the dosage of a drug. In these cases, the lowest commercially available dosage strength of a tablet was considered to estimate the minimum amount taken to ensure that an overdose actually occurred and not “just” several tablets of the lowest strength were taken within the MDD.

MDD was determined on the basis of the prescribing information, whereby a high MDD was presumed for the clear detection of actual intoxication. However, this procedure allows a certain degree of discretion on the part of those conducting the study. In addition, substances such as benzodiazepines or opioids are subject to a strong habituation effect, so that no standardized MDD can be determined.

Furthermore, as our survey was limited to the inpatient-somatic stay cases, a long-term follow-up of patients is lacking.

Finally, since the data collection was not hypothesis-driven, but it was rather aimed at capturing the interrelationships of a variety of parameters, random findings may have arisen due to multiple testing. Therefore, statistically significant results should be interpreted as trends until they will be validated in separate confirmatory studies.

## Conclusions

The present study provides both a broad spectrum of descriptive data and a comprehensive presentation of person-, substance- and intoxication profiles of self-poisoning patients with SRB. The results of this work confirm known patterns and risk factors of SRB by intoxication and underline the complexity of (para-)suicidal behavior. Due to the multifactorial genesis of (para-)suicidal intoxications, there are many potential starting points for their prevention, such as restricting access to certain frequently used substances or also preclinically identifying particular at-risk groups and providing them in advance with appropriate preventive assistance measures.

## Electronic supplementary material

Below is the link to the electronic supplementary material.

Supplementary file1 (DOCX 44 KB)

Supplementary file2 (DOCX 35 KB)

Supplementary file3 (DOCX 54 KB)

Supplementary file4 (DOCX 34 KB)

## Data Availability

Data is provided within the manuscript or supplementary information files.
