# Supplementary material for: Clinical course and demographic insights into suicide by self-poisoning: patterns of substance use and socio-economic factors
Source: Soc Psychiatry Psychiatr Epidemiol. 2024 Sep 24;60(3):705–18. doi: 10.1007/s00127-024-02750-x (PMC11870874; doi:10.1007/s00127-024-02750-x)
Supplement: Supplementary file 1 — Supplementary file1 (DOCX 44 KB) [file 127_2024_2750_MOESM1_ESM.docx]

**Online Resource Table 1** Maximum daily doses (MDD) and grouping of the individual substances into substance classes

| **Medication** | **Substance class** | **MDD** | **Unit** |
| --- | --- | --- | --- |
| Agomelatine | Antidepressant | 50 | mg |
| Alprazolam | Benzodiazepine | 3 | mg |
| Ambroxol | Other drugs | 60 | mg |
| Amisulpride | Antipsychotic | 1200 | mg |
| Amitriptyline | Antidepressant | 300 | mg |
| Amitriptyline retard | Antidepressant | 300 | mg |
| Amlodipine | Cardiac medication | 10 | mg |
| Amoxicillin | Antibiotic | 3000 | mg |
| Aripiprazole | Antipsychotic | 30 | mg |
| Aspirin (ASS) | Non-opioid analgesics | 3000 | mg |
| Atenolol | Cardiac medication | 100 | mg |
| Atorvastatin | Endocrinological medication | 80 | mg |
| Valerian | Herbal medicine | / | mg |
| Benazepril | Cardiac medication | 40 | mg |
| Benserazide | Anti-Parkinson-medication | 200 | mg |
| Biperiden | Anti-Parkinson-medication | 16 | mg |
| Bisoprolol | Cardiac medication | 20 | mg |
| Bromazepam | Benzodiazepine | 18 | mg |
| Bromocriptine | Anti-Parkinson-medication | 20 | mg |
| Bromperidol | Antipsychotic | 50 | mg |
| Brotizolam | Benzodiazepine | / | mg |
| Buprenorphine | Opioid | 8 | mg |
| Bupropion | Antidepressant | 300 | mg |
| Butylscopolamine | Other drugs | 60 | mg |
| Candesartan | Cardiac medication | 32 | mg |
| Carbamazepine | Anticonvulsant | 1600 | mg |
| Carbimazole | Endocrinological medication | 60 | mg |
| Carvedilol | Cardiac medication | 50 | mg |
| Cefuroxime | Antibiotic | 1000 | mg |
| Celecoxib | Non-opioid analgesics | 400 | mg |
| Cetirizine | Antihistamine | 20 | mg |
| Chloral hydrate | Other sedatives | 1500 | mg |
| Chloroquine | Other drugs | 500 | mg |
| Chlorprothixene | Antipsychotic | 400 | mg |
| Ciprofloxacin | Antibiotic | 750 | mg |
| Citalopram | Antidepressant | 40 | mg |
| Clarithromycin | Antibiotic | 1000 | mg |
| Clindamycin | Antibiotic | 1800 | mg |
| Clomethiazole | Other sedatives | 768 | mg |
| Clomipramine | Antidepressant | 150 | mg |
| Clonazepam | Benzodiazepine | 8 | mg |
| Clopidogrel | Anticoagulant | 75 | mg |
| Clorazepate | Benzodiazepine | 300 | mg |
| Clozapine | Antipsychotic | 900 | mg |
| Codeine | Opioid | 200 | mg |
| Caffeine | Other drugs | 400 | mg |
| Colchicine | Other drugs | 12 | mg |
| Cholecalciferol | Other drugs | / | mg |
| Trimethoprim/sulfamethoxazole (Cotrimoxazole) | Antibiotic | 1920 | mg |
| Desloratadine | Antihistamine | 5 | mg |
| Dextromethorphan | Other drugs | 120 | mg |
| Diazepam | Benzodiazepine | 60 | mg |
| Diclofenac | Non-opioid analgesics | 150 | mg |
| Diethylamine propiophenone | Other drugs | / | mg |
| Digitoxin | Cardiac medication | 0.3 | mg |
| Digoxin | Cardiac medication | 1.2 | mg |
| Dimenhydrinate | Antihistamine | 400 | mg |
| Diphenhydramine | Antihistamine | 50 | mg |
| Domperidone | Other drugs | 30 | mg |
| Doxazosin | Cardiac medication | 16 | mg |
| Doxepin | Antidepressant | 300 | mg |
| Doxycycline | Antibiotic | 200 | mg |
| Doxylamine | Antihistamine | 50 | mg |
| Drotaverine | Other drugs | 240 | mg |
| Duloxetine | Antidepressant | 120 | mg |
| Iron | Other drugs | 300 | mg |
| Escitalopram | Antidepressant | 20 | mg |
| Esomeprazole | Other drugs | 160 | mg |
| Etoricoxib | Non-opioid analgesics | 120 | mg |
| Fentanyl | Opioid | / | mg |
| Fexofenadine | Antihistamine | 180 | mg |
| Flunitrazepam | Benzodiazepine | 2 | mg |
| Fluoxetine | Antidepressant | 60 | mg |
| Flupentixol | Antipsychotic | 60 | mg |
| Flupirtine | Non-opioid analgesics | 400 | mg |
| Flurazepam | Benzodiazepine | 30 | mg |
| Frovatriptan | Other drugs | 5 | mg |
| Furosemide | Cardiac medication | 1000 | mg |
| Gabapentin | Anticonvulsant | 3600 | mg |
| Haloperidol | Antipsychotic | 100 | mg |
| Hydrochlorothiazide (HCT) | Cardiac medication | 100 | mg |
| Yeast extract | Other substances | / | mg |
| Human insulin | Antidiabetic | / | mg |
| Hydromorphone | Opioid | / | mg |
| Hydroxyzine | Antihistamine | 100 | mg |
| Ibuprofen | Non-opioid analgesics | 2400 | mg |
| Imipramine | Antidepressant | 300 | mg |
| Insulin | Antidiabetic | / | mg |
| Irbesartan | Cardiac medication | 300 | mg |
| Isoniazid | Antibiotic | 300 | mg |
| Itraconazole | Fungicide | 400 | mg |
| Ivabradine | Cardiac medication | 15 | mg |
| Iodine | Other drugs | 200 | mg |
| Potassium | Other drugs | / | mg |
| Ketoprofen | Non-opioid analgesics | 200 | mg |
| L-Thyroxine / Levothyroxine | Endocrinological medication | 300 | µg |
| Lamotrigine | Anticonvulsant | 700 | mg |
| Lercanidipine | Cardiac medication | 20 | mg |
| Levetiracetam | Anticonvulsant | 3000 | mg |
| Levocetirizine | Antihistamine | 5 | mg |
| Levodopa | Anti-Parkinson-medication | 800 | mg |
| Levomepromazine | Antipsychotic | 500 | mg |
| Lidocaine | Other drugs | / | mg |
| Lisinopril | Cardiac medication | 20 | mg |
| Lithium | Antidepressant | / | mg |
| Lithium carbonate / Lithium retard | Antidepressant | 1350 | mg |
| Loperamide | Other drugs | 16 | mg |
| Lorazepam | Benzodiazepine | 7.5 | mg |
| Lormetazepam | Benzodiazepine | 2 | mg |
| Losartan | Cardiac medication | 150 | mg |
| Magnesium | Other drugs | / | mg |
| Warfarin (Marcumar) | Anticoagulant | 9 | mg |
| Melperone | Antipsychotic | 400 | mg |
| Metamizole | Non-opioid analgesics | 4000 | mg |
| Metformin | Antidiabetic | 3000 | mg |
| Methocarbamol | Other drugs | 7500 | mg |
| Methotrexate | Cytostatic | / | mg |
| Methylphenidate | Other drugs | 80 | mg |
| Metoclopramide | Other drugs | 30 | mg |
| Metoprolol | Cardiac medication | 200 | mg |
| Metronidazole | Antibiotic | 2000 | mg |
| Mianserin | Antidepressant | 90 | mg |
| Midazolam | Benzodiazepine | / | mg |
| Mirtazapine | Antidepressant | 45 | mg |
| Moclobemide | Antidepressant | 600 | mg |
| Molsidomine | Cardiac medication | 16 | mg |
| Morphine | Opioid | 360 | mg |
| Morphine retard | Opioid | 150 | mg |
| Naloxone | Other drugs | / | mg |
| Naltrexone | Other drugs | 50 | mg |
| Naproxen | Non-opioid analgesics | 600 | mg |
| Nebivolol | Cardiac medication | 5 | mg |
| Nitrazepam | Benzodiazepine | 10 | mg |
| Nortriptyline | Antidepressant | 225 | mg |
| Olanzapine | Antipsychotic | 20 | mg |
| Olmesartan | Cardiac medication | 40 | mg |
| Omeprazole | Other drugs | 120 | mg |
| Opipramol | Antidepressant | 300 | mg |
| Oxazepam | Benzodiazepine | 200 | mg |
| Oxycodone | Opioid | 400 | mg |
| Pantoprazole | Other drugs | 160 | mg |
| Paracetamol | Non-opioid analgesics | 4000 | mg |
| Paroxetine | Antidepressant | 60 | mg |
| Penicillin | Antibiotic | / | mg |
| Penicillin G | Antibiotic | / | mg |
| Perazine | Antipsychotic | 1000 | mg |
| Phenobarbital | Anticonvulsant | / | mg |
| Phenprocoumon | Anticoagulant | 9 | mg |
| Pipamperone | Antipsychotic | 360 | mg |
| Pregabalin | Anticonvulsant | 600 | mg |
| Pridinol | Other drugs | 12 | mg |
| Promethazine | Antipsychotic | 200 | mg |
| Propofol | Other sedatives | / | mg |
| Propranolol | Cardiac medication | 320 | mg |
| Prothipendyl | Antipsychotic | 320 | mg |
| Quetiapine / Quetiapine retard | Antipsychotic | 800 | mg |
| Ramipril | Cardiac medication | 10 | mg |
| Ranitidine | Other drugs | 900 | mg |
| Risperidone | Antipsychotic | 10 | mg |
| Rivaroxaban | Anticoagulant | 30 | mg |
| Sertraline | Antidepressant | 200 | mg |
| Simvastatin | Endocrinological medication | 80 | mg |
| Sinupret | Herbal medicine | / | mg |
| Sulpiride | Antipsychotic | 1600 | mg |
| Tamsulosin | Other drugs | 0.4 | mg |
| Tapentadol | Opioid | 700 | mg |
| Terbinafine | Fungicide | 250 | mg |
| Terfenadine | Antihistamine | 120 | mg |
| Tetrazepam | Benzodiazepine | 400 | mg |
| Theophylline | Other drugs | / | mg |
| Tilidine | Opioid | 600 | mg |
| Tizanidine | Other drugs | / | mg |
| Tolperisone | Other drugs | 450 | mg |
| Tolterodine | Other drugs | 4 | mg |
| Topiramate | Anticonvulsant | 400 | mg |
| Torasemide | Cardiac medication | 200 | mg |
| Tramadol | Opioid | 400 | mg |
| Tranylcypromine | Antidepressant | 60 | mg |
| Trazodone | Antidepressant | 600 | mg |
| Triazolam | Benzodiazepine | 0.25 | mg |
| Trimipramine | Antidepressant | 400 | mg |
| Urapidil | Cardiac medication | / | mg |
| Valproate | Anticonvulsant | 2500 | mg |
| Valsartan | Cardiac medication | 320 | mg |
| Venlafaxine | Antidepressant | 375 | mg |
| Verapamil | Cardiac medication | 480 | mg |
| Xipamide | Cardiac medication | 80 | mg |
| Ziprasidone | Antipsychotic | 160 | mg |
| Zolpidem | Z-Drug | 10 | mg |
| Zopiclone | Z-Drug | 7.5 | mg |
| Zuclopenthixol | Antipsychotic | 150 | mg |
