# Supplementary material for: Clinical course and demographic insights into suicide by self-poisoning: patterns of substance use and socio-economic factors
Source: Soc Psychiatry Psychiatr Epidemiol. 2024 Sep 24;60(3):705–18. doi: 10.1007/s00127-024-02750-x (PMC11870874; doi:10.1007/s00127-024-02750-x)
Supplement: Supplementary file 2 — Supplementary file2 (DOCX 35 KB) [file 127_2024_2750_MOESM2_ESM.docx]

**Online Resource Table 2** Patient characteristics

| **Characteristic** |  |  | **n (%)** |
| --- | --- | --- | --- |
| **Sociodemographic data** | Nationality | German | 323 (65.1) |
|  |  | Afghan | 16 (3.2) |
|  |  | Turkish | 9 (1.8) |
|  |  | Polish | 8 (1.6) |
|  |  | Iraqi | 7 (1.4) |
|  |  | Croatian | 5 (1.0) |
|  |  | Other 44 nationalities | 128 (25.8) |
|  |  | Missing | 594 |
| **Anamnestic data** | Somatic diseases | Cardiovascular | 148 (13.6) |
|  |  | Pulmonary | 60 (5.5) |
|  |  | Neurological | 147 (13.5) |
|  |  | Neoplastic | 43 (3.9) |
|  |  | Infectious | 23 (2.1) |
|  |  | Metabolic | 149 (13.7) |
| **Preclinical data** | Rescue person | Relative/friend/neighbor | 659 (69.2) |
|  |  | Patient himself | 167 (17.5) |
|  |  | Facility staff | 69 (7.2) |
|  |  | Passerby | 37 (3.9) |
|  |  | Police/Security | 20 (2.1) |
|  |  | Missing | 138 |
|  | Rescue time | <1h | 84 (11.9) |
|  |  | 1-3h | 322 (45.7) |
|  |  | 3-6h | 97 (13.8) |
|  |  | >6h | 202 (28.7) |
|  |  | Missing | 385 |
|  | Initial GCS | 15 | 473 (54.9) |
|  |  | 14 | 89 (10.3) |
|  |  | 13 | 57 (6.6) |
|  |  | 12 | 33 (3.8) |
|  |  | 11 | 41 (4.8) |
|  |  | 10 | 26 (3.0) |
|  |  | 9 | 25 (2.9) |
|  |  | 8 | 25 (2.9) |
|  |  | 7 | 18 (2.1) |
|  |  | 6 | 14 (1.6) |
|  |  | 5 | 4 (0.5) |
|  |  | 4 | 14 (1.6) |
|  |  | 3 | 42 (4.9) |
|  |  | Missing | 229 |
| **Clinical data** | Inpatient length of stay | ≤24h | 320 (29.4) |
|  |  | 25-48h | 224 (20.6) |
|  |  | 49-72h | 167 (15.3) |
|  |  | 73-96h | 97 (8.9) |
|  |  | ≥97h | 282 (25.9) |
|  | Activated charcoal |  | 97 (9.0) |
|  | Antidote therapy |  | 178 (16.3) |
|  | Mechanical ventilation |  | 121 (11.1) |
|  | Hemodialysis |  | 24 (2.2) |
|  | Number of substances ingested | 1 | 595 (54.8) |
|  |  | 2 | 239 (22.00) |
|  |  | 3 | 133 (12.2) |
|  |  | >3 | 119 (11.0) |
|  |  | missing | 4 |

*Data are in n (%) unless otherwise noted. Due to rounding, percentages may not add up to 100%.*
