# Supplementary material for: Clinical course and demographic insights into suicide by self-poisoning: patterns of substance use and socio-economic factors
Source: Soc Psychiatry Psychiatr Epidemiol. 2024 Sep 24;60(3):705–18. doi: 10.1007/s00127-024-02750-x (PMC11870874; doi:10.1007/s00127-024-02750-x)
Supplement: Supplementary file 3 — Supplementary file3 (DOCX 54 KB) [file 127_2024_2750_MOESM3_ESM.docx]

**Online Resource Table 3** Frequencies of the substance categories by sex and age group

| **Substance categories*** | **Total**  **n=1090** | **Male**  **n=363** | **Female**  **n=727** | **p-value** | **<18**  **n=58** | **18-44 n=603** | **45-64 n=318** | **>64**  **n=111** | **p-value** |
| --- | --- | --- | --- | --- | --- | --- | --- | --- | --- |
| **Antibiotic** | **22 (2.0)** | **5 (1.4)** | **17 (2.3)** | **0.364** | **3 (5.2)** | **19 (3.2)** | **0 (0)** | **0 (0)** | **<0.001** |
| Single ingestion | 3 (13.6) | 1 (20.0) | 2 (11.8) |  | 1 (33.3) | 2 (10.5) | 0 (0.0) | 0 (0.0) |  |
| Multiple ingestion | 19 (86.4) | 4 (80.0) | 15 (88.2) |  | 2 (66.7) | 17 (89.5) | 0 (0.0) | 0 (0.0) |  |
| **Anticoagulant** | **9 (1.0)** | **7 (2.0)** | **2 (0.3)** | **0.008** | **0 (0)** | **2 (0.3)** | **5 (1.6)** | **2 (1.8)** | **0.096** |
| Single ingestion | 2 (22.2) | 2 (28.6) | 0 (0.0) |  | 0 (0.0) | 0 (0.0) | 2 (40.0) | 0 (0.0) |  |
| Multiple ingestion | 7 (77.8) | 5 (71.4) | 2 (100.0) |  | 0 (0.0) | 2 (100.0) | 3 (60.0) | 2 (100.0) |  |
| **Anticonvulsant** | **94 (8.6)** | **34 (9.4)** | **60 (8.3)** | **0.567** | **6 (10.3)** | **54 (9.0)** | **22 (6.9)** | **12 (10.8)** | **0.55** |
| Single ingestion | 26 (27.7) | 12 (35.3) | 14 (23.3) |  | 4 (66.7) | 10 (18.5) | 8 (36.4) | 4 (33.3) |  |
| Multiple ingestion | 68 (72.3) | 22 (64.7) | 46 (76.7) |  | 2 (33.3) | 44 (81.5) | 14 (63.6) | 8 (66.7) |  |
| **Antidepressant** | **308 (28.3)** | **85 (23.4)** | **223 (30.7)** | **0.013** | **15 (25.9)** | **175 (29.0)** | **97 (30.5)** | **21 (18.9)** | **0.116** |
| Single ingestion | 103 (33.4) | 30 (35.3) | 73 (32.7) |  | 8 (53.3) | 58 (33.1) | 32 (33.0) | 5 (23.8) |  |
| Multiple ingestion | 205 (66.6) | 55 (64.7) | 150 (67.3) |  | 7 (46.7) | 117 (66.9) | 65 (67.0) | 16 (76.2) |  |
| **Antidiabetic** | **13 (1.2)** | **4 (1.1)** | **9 (1.2)** | **1.00** | **0 (0)** | **5 (0.8)** | **5 (1.6)** | **3 (2.7)** | **0.261** |
| Single ingestion | 4 (30.8) | 1 (25.0) | 3 (33.3) |  | 0 (0.0) | 2 (40.0) | 1 (20.0) | 1 (33.3) |  |
| Multiple ingestion | 9 (69.2) | 3 (75.0) | 6 (66.7) |  | 0 (0.0) | 3 (60.0) | 4 (80.0) | 2 (66.7) |  |
| **Antihistamine** | **76 (7.0)** | **21 (5.8)** | **55 (7.6)** | **0.314** | **1 (1.7)** | **55 (9.1)** | **16 (5.0)** | **4 (3.6)** | **0.017** |
| Single ingestion | 32 (42.1) | 12 (57.1) | 20 (36.4) |  | 1 (100.0) | 20 (36.4) | 10 (62.5) | 1 (25.0) |  |
| Multiple ingestion | 44 (57.9) | 9 (42.9) | 35 (63.6) |  | 0 (0.0) | 35 (63.6) | 37.5) | 3 (75.0) |  |
| **Anti-Parkinson-medication** | **9 (1.0)** | **2 (0.6)** | **7 (1.0)** | **0.726** | **0 (0)** | **5 (0.8)** | **1 (0.3)** | **3 (2.7)** | **0.156** |
| Single ingestion | 0 (0.0) | 0 (0.0) | 0 (0.0) |  | 0 (0.0) | 0 (0.0) | 0 (0.0) | 0 (0.0) |  |
| Multiple ingestion | 9 (100.0) | 2 (100.0) | 7 (100.0) |  | 0 (0.0) | 5 (100.0) | 1 (100.0) | 3 (100.0) |  |
| **Antipsychotic** | **191 (17.5)** | **65 (17.9)** | **126 (17.3)** | **0.866** | **5 (8.6)** | **112 (18.6)** | **62 (19.5)** | **12 (10.8)** | **0.047** |
| Single ingestion | 54 (28.3) | 19 (29.2) | 35 (27.8) |  | 4 (80.0) | 31 (27.7) | 18 (29.0) | 1 (8.3) |  |
| Multiple ingestion | 137 (71.7) | 46 (70.8) | 91 (72.2) |  | 1 (20.0) | 81 (72.3) | (71.0) | 11 (91.7) |  |
| **Benzodiazepine** | **267 (24.5)** | **65 (17.9)** | **202 (27.8)** | **<0.001** | **4 (6.9)** | **147 (24.4)** | **83 (26.1)** | **33 (29.7)** | **0.003** |
| Single ingestion | 98 (36.7) | 22 (33.8) | 76 (37.6) |  | 1 (25.0) | 47 (32.0) | 37 (44.6) | 13 (39.4) |  |
| Multiple ingestion | 169 (63.3) | 43 (66.2) | 126 (62.4) |  | 3 (75.0) | 100 (68.0) | 46 (55.4) | 20 (60.6) |  |
| **Car exhaust/**  **carbon monoxide** | **20 (1.8)** | **18 (5.0)** | **2 (0.3)** | **<0.001** | **0 (0)** | **10 (1.7)** | **8 (2.5)** | **2 (1.8)** | **0.705** |
| Single ingestion | 18 (90.0) | 16 (88.9) | 2 (100.0) |  | 0 (0.0) | 9 (90.0) | 7 (87.5) | 2 (100.0) |  |
| Multiple ingestion | 2 (10.0) | 2 (11.1) | 0 (0.0) |  | 0 (0.0) | 1 (10.0) | 1 (12.5) | 0 (0.0) |  |
| **Cardiac medication** | **67 (6.1)** | **33 (9.1)** | **34 (4.7)** | **0.005** | **2 (3.4)** | **26 (4.3)** | **28 (8.8)** | **11 (9.9)** | **0.012** |
| Single ingestion | 10 (14.9) | 6 (18.2) | 4 (11.8) |  | 2 (100.0) | 1 (3.8) | 6 (21.4) | 1 (9.1) |  |
| Multiple ingestion | 57 (85.1) | 27 (81.8) | 30 (88.2) |  | 0 (0.0) | 25 (96.2) | 22 (78.6) | 10 (90.9) |  |
| **Chemical** | **11 (1.0)** | **6 (1.7)** | **5 (0.7)** | **0.195** | **1 (1.7)** | **4 (0.7)** | **6 (1.9)** | **0 (0)** | **0.177** |
| Single ingestion | 9 (81.8) | 6 (100.0) | 3 (60.0) |  | 1 (100.0) | 3 (75.0) | 5 (83.3) | 0 (0.0) |  |
| Multiple ingestion | 2 (18.2) | 0 (0.0) | 2 (40.0) |  | 0 (0.0) | 1 (25.0) | 1 (16.7) | 0 (0.0) |  |
| **Cleaning agents** | **19 (1.7)** | **8 (2.2)** | **11 (1.5)** | **0.463** | **1 (1.7)** | **12 (2.0)** | **2 (0.6)** | **4 (3.6)** | **0.123** |
| Single ingestion | 16 (84.2) | 6 (75.0) | 10 (90.9) |  | 1 (100.0) | 9 (75.0) | 2 (100.0) | 4 (100.0) |  |
| Multiple ingestion | 3 (15.8) | 2 (25.0) | 1 (9.1) |  | 0 (0.0) | 3 (25.0) | 0 (0.0) | 0 (0.0) |  |
| **Cytostatic** | **3 (0.3)** | **1 (0.3)** | **2 (0.3)** | **1.000** | **0 (0)** | **1 (0.2)** | **2 (0.6)** | **0 (0)** | **0.563** |
| Single ingestion | 2 (66.7) | 0 (0.0) | 2 (100.0) |  | 0 (0.0) | 1 (100.0) | 1 (50.0) | 0 (0.0) |  |
| Multiple ingestion | 1 (33.3) | 1 (100.0) | 0 (0.0) |  | 0 (0.0) | 0 (0.0) | 1 (50.0) | 0 (0.0) |  |
| **Endocrinological medication** | **16 (1.5)** | **9 (2.5)** | **7 (1.0)** | **0.062** | **0 (0)** | **7 (1.2)** | **5 (1.6)** | **4 (3.6)** | **0.223** |
| Single ingestion | 0 (0.0) | 0 (0.0) | 0 (0.0) |  | 0 (0.0) | 0 (0.0) | 0 (0.0) | 0 (0.0) |  |
| Multiple ingestion | 16 (100.0) | 9 (100.0) | 7 (100.0) |  | 0 (0.0) | 7 (100.0) | 5 (100.0) | 4 (100.0) |  |
| **Fungicide** | **3 (0.3)** | **1 (0.3)** | **2 (0.3)** | **1.000** | **0 (0)** | **2 (0.3)** | **1 (0.3)** | **0 (0)** | **1** |
| Single ingestion | 1 (33.3) | 0 (0.0) | 1 (50.0) |  | 0 (0.0) | 1 (50.0) | 0 (0.0) | 0 (0.0) |  |
| Multiple ingestion | 2 (66.7) | 1 (100.0) | 1 (50.0) |  | 0 (0.0) | 1 (50.0) | 1 (100.0) | 0 (0.0) |  |
| **Herbal medicine** | **18 (1.7)** | **4 (1.1)** | **14 (1.9)** | **0.451** | **1 (1.7)** | **14 (2.3)** | **3 (0.9)** | **0 (0)** | **0.21** |
| Single ingestion | 1 (5.6) | 1 (25.0) | 0 (0.0) |  | 0 (0.0) | 1 (7.1) | 0 (0.0) | 0 (0.0) |  |
| Multiple ingestion | 17 (94.4) | 3 (75.0) | 14 (100.0) |  | 1 (100.0) | 13 (92.9) | 3 (100.0) | 0 (0.0) |  |
| **(Illegal) drugs** | **42 (3.9)** | **19 (5.2)** | **23 (3.2)** | **0.098** | **7 (12.1)** | **23 (4.0)** | **8 (2.5)** | **4 (3.6)** | **0.02** |
| Single ingestion | 28 (66.7) | 13 (68.4) | 15 (65.2) |  | 5 (71.4) | 12 (52.2) | 7 (87.5) | 4 (100.0) |  |
| Multiple ingestion | 14 (33.3) | 6 (31.6) | 8 (34.8) |  | 2 (28.6) | 11 (47.8) | 1 (12.5) | 0 (0.0) |  |
| **Insecticide** | **6 (0.6)** | **5 (1.4)** | **1 (0.1)** | **0.017** | **0 (0)** | **3 (0.5)** | **2 (0.6)** | **1 (0.9)** | **0.79** |
| Single ingestion | 5 (83.3) | 5 (100.0) | 0 (0.0) |  | 0 (0.0) | 3 (100.0) | 1 (50.0) | 1 (100.0) |  |
| Multiple ingestion | 1 (16.7) | 0 (0.0) | 1 (100.0) |  | 0 (0.0) | 0 (0.0) | 1 (50.0) | 0 (0.0) |  |
| **Mushrooms** | **1 (0.1)** | **1 (0.3)** | **0 (0)** | **0.333** | **0 (0)** | **0 (0)** | **0 (0)** | **1 (0.9)** | **0.155** |
| Single ingestion | 1 (100.0) | 1 (100.0) | 0 (0.0) |  | 0 (0.0) | 0 (0.0) | 0 (0.0) | 1 (100.0) |  |
| Multiple ingestion | 0 (0.0) | 0 (0.0) | 0 (0.0) |  | 0 (0.0) | 0 (0.0) | 0 (0.0) | 0 (0.0) |  |
| **Non-opioid analgesics** | **264 (24.4)** | **77 (21.2)** | **187 (25.7)** | **0.115** | **23 (39.7)** | **169 (28.0)** | **52 (16.4)** | **20 (18.0)** | **<0.001** |
| Single ingestion | 80 (30.3) | 23 (29.9) | 57 (30.5) |  | 8 (34.8) | 57 (33.7) | 13 (25.0) | 2 (10.0) |  |
| Multiple ingestion | 184 (69.7) | 54 (70.1) | 130 (69.5) |  | 15 (65.2) | 112 (66.3) | 39 (75.0) | 18 (90.0) |  |
| **Opioids** | **74 (6.8)** | **23 (6.3)** | **51 (7.0)** | **0.704** | **1 (1.7)** | **40 (6.6)** | **20 (6.3)** | **13 (11.7)** | **0.096** |
| Single ingestion | 9 (12.2) | 2 (8.7) | 7 (13.7) |  | 0 (0.0) | 6 (15.0) | 2 (10.0) | 1 (7.7) |  |
| Multiple ingestion | 65 (87.8) | 21 (91.3) | 44 (86.3) |  | 1 (100.0) | 34 (85.0) | 18 (90.0) | 12 (92.3) |  |
| **Other drugs** | **81 (7.4)** | **22 (6.1)** | **59 (8.1)** | **0.270** | **5 (8.6)** | **54 (9.0)** | **18 (5.7)** | **4 (3.6)** | **0.11** |
| Single ingestion | 16 (19.8) | 4 (18.2) | 12 (20.3) |  | 1 (20.0) | 11 (20.4) | 4 (22.2) | 0 (0.0) |  |
| Multiple ingestion | 65 (80.2) | 18 (81.8) | 47 (79.7) |  | 4 (80.0) | 43 (79.6) | 14 (77.8) | 4 (100.0) |  |
| **Other sedatives** | **7 (0.6)** | **5 (1.4)** | **2 (0.3)** | **0.045** | **0 (0)** | **5 (0.8)** | **1 (0.3)** | **1 (0.9)** | **0.74** |
| Single ingestion | 6 (85.7) | 4 (80.0) | 2 (100.0) |  | 0 (0.0) | 4 (80.0) | 1 (100.0) | 1 (100.0) |  |
| Multiple ingestion | 1 (14.3) | 1 (20.0) | 0 (0.0) |  | 0 (0.0) | 1 (20.0) | 0 (0.0) | 0 (0.0) |  |
| **Other substances** | **4 (0.4)** | **2 (0.6** | **2 (0.3)** | **0.604** | **1 (1.7)** | **2 (0.3)** | **1 (0.3)** | **0 (0)** | **0.316** |
| Single ingestion | 3 (75.0) | 2 (100.0) | 1 (50.0) |  | 0 (0.0) | 2 (100.0) | 1 (100.0) | 0 (0.0) |  |
| Multiple ingestion | 1 (25.0) | 0 (0.0) | 1 (50.0) |  | 1 (100.0) | 0 (0.0) | 0 (0.0) | 0 (0.0) |  |
| **Plant** | **7 (0.6)** | **5 (1.4)** | **2 (0.3)** | **0.045** | **0 (0)** | **6 (1.0)** | **1 (0.3)** | **0 (0)** | **0.615** |
| Single ingestion | 5 (71.4) | 3 (60.0) | 2 (100.0) |  | 0 (0.0) | 4 (66.7) | 1 (100.0) | 0 (0.0) |  |
| Multiple ingestion | 2 (28.6) | 2 (40.0) | 0 (0.0) |  | 0 (0.0) | 2 (33.3) | 0 (0.0) | 0 (0.0) |  |
| **Rodenticides** | **5 (0.5)** | **3 (0.8)** | **2 (0.3)** | **0.340** | **0 (0)** | **2 (0.3)** | **3 (0.9)** | **0 (0)** | **0.538** |
| Single ingestion | 5 (100.0) | 3 (100.0) | 2 (100.0) |  | 0 (0.0) | 2 (100.0) | 3 (100.0) | 0 (0.0) |  |
| Multiple ingestion | 0 (0.0) | 0 (0.0) | 0 (0.0) |  | 0 (0.0) | 0 (0.0) | 0 (0.0) | 0 (0.0) |  |
| **Z-drugs** | **147 (13.5)** | **40 (11.0)** | **107 (14.7)** | **0.109** | **1 (1.7)** | **61 (10.1)** | **48 (15.1)** | **37 (33.3)** | **<0.001** |
| Single ingestion | 58 (39.5) | 16 (40.0) | 42 (39.3) |  | 1 (100.0) | 22 (36.1) | 18 (37.5) | 17 (45.9) |  |
| Multiple ingestion | 89 (60.5) | 24 (60.0) | 65 (60.7) |  | 0 (0.0) | 39 (63.9) | 30 (62.5) | 20 (54.1) |  |

**Each substance category was recorded only once per patient. Data are in n (%) unless otherwise noted. Due to rounding, percentages may not add up to 100%.*
