# Supplementary material for: Clinical course and demographic insights into suicide by self-poisoning: patterns of substance use and socio-economic factors
Source: Soc Psychiatry Psychiatr Epidemiol. 2024 Sep 24;60(3):705–18. doi: 10.1007/s00127-024-02750-x (PMC11870874; doi:10.1007/s00127-024-02750-x)
Supplement: Supplementary file 4 — Supplementary file4 (DOCX 34 KB) [file 127_2024_2750_MOESM4_ESM.docx]

**Online Resource Fig. 1** Study flowchart
